# The Impact of Smoking on Subgingival Microflora: From Periodontal Health to Disease

**DOI:** 10.3389/fmicb.2020.00066

**Published:** 2020-01-29

**Authors:** Yaling Jiang, Xuedong Zhou, Lei Cheng, Mingyun Li

**Affiliations:** ^1^State Key Laboratory of Oral Diseases, National Clinical Research Center for Oral Diseases, West China Hospital of Stomatology, Sichuan University, Chengdu, China; ^2^Department of Operative Dentistry and Endodontics, West China Hospital of Stomatology, Sichuan University, Chengdu, China

**Keywords:** periodontal disease, smoking, subgingival microflora, nicotine, microbial diversity

## Abstract

Periodontal disease is one of the most common diseases of the oral cavity affecting up to 90% of the worldwide population. Smoking has been identified as a major risk factor in the development and progression of periodontal disease. It is essential to assess the influence of smoking on subgingival microflora that is the principal etiological factor of the disease to clarify the contribution of smoking to periodontal disease. Therefore, this article reviews the current research findings regarding the impact of smoking on subgingival microflora and discusses several potential mechanisms. Cultivation-based and targeted molecular approaches yield controversial results in determining the presence or absence of smoking-induced differences in the prevalence or levels of certain periodontal pathogens, such as the “red complex.” However, substantial changes in the subgingival microflora of smokers, regardless of their periodontal condition (clinical health, gingivitis, or periodontitis), have been demonstrated in recent microbiome studies. Available literature suggests that smoking facilitates early acquisition and colonization of periodontal pathogens, resulting in an “at-risk-for-harm” subgingival microbial community in the healthy periodontium. In periodontal diseases, the subgingival microflora in smokers is characterized by a pathogen-enriched community with lower resilience compared to that in non-smokers, which increases the difficulty of treatment. Biological changes in key pathogens, such as *Porphyromonas gingivalis*, together with the ineffective host immune response for clearance, might contribute to alterations in the subgingival microflora in smokers. Nonetheless, further studies are necessary to provide solid evidence for the underlying mechanisms.

## Introduction

Smoking remains a highly prevalent addiction in many populations worldwide despite the increasing awareness of its harmful effects on general health (World Health Organization [WHO], 2018). The number of smokers is >1.1 billion (1 out of 7) globally now, and over 8 million people die annually because of smoking^1^. As one of the five leading risk factors for the global burden of the disease, smoking is responsible for various diseases, including cancer, cardiovascular disease, chronic obstructive pulmonary disease, and periodontal disease (GBD 2016 Risk Factors Collaborators, 2017).

Periodontal disease, also known as gum disease, comprises a range of polymicrobial infectious disorders (such as gingivitis and periodontitis) that affect the tooth-supporting tissues (the gingiva, alveolar bone, and periodontal ligament). It is the most common cause of tooth loss and also contributes to systemic diseases. Smoking has been recognized as a major risk factor for periodontal disease, affecting the prevalence, severity, progression, and treatment response of the disease, second only to the dental plaque. Epidemiological studies have presented a significantly higher risk for periodontal disease in smokers compared to non-smokers, and the increased risk is proportional to the duration and rate of smoking (Do et al., 2008; Bergstrom, 2014; Eke et al., 2016). Smokers exhibit a clinically distinct predisposition to periodontal disease, with deeper pockets, more extensive and severe attachment loss, greater levels of bone destruction, and a higher rate of tooth loss (Ylostalo et al., 2004; Baljoon et al., 2005; Johnson and Guthmiller, 2007). In addition, smoking exerts an adverse influence on the clinical treatment outcomes of non-surgical and surgical therapies as well as the long-term success of implant placement (Heasman et al., 2006; Patel et al., 2012).

Considering the well-established deleterious effects of smoking on periodontal health, it is of great importance to understand the underlying mechanisms, which remain largely unclear. Its widely accepted that both periodontal microflora and host response play critical roles in the initiation and progression of periodontal disease (Kinane et al., 2017). Considerable attention has been focused on the effects of smoking on host response in previous studies, which demonstrate that smoking increases the host’s susceptibility and risk of infection by inducing immune dysfunction (Ryder, 2007; Lee et al., 2012). However, it is still necessary to carry out a more detailed assessment of the effects of smoking on subgingival microflora that causes the infectious disease. A previous review article investigated the correlation between smoking and oral and nasopharyngeal bacterial flora, and demonstrated the adverse effects of smoking on the colonization of potential pathogens and the increased frequency of upper respiratory tract infections (Brook, 2011). This review aims to present current research findings of the impact of smoking on subgingival microflora and discuss possible mechanisms by which smoking interferes with the microflora.

## Smoking and Subgingival Microflora

### *In vitro* Effects of Smoking on Key Periodontal Pathogens

#### Model Pathogens and Proxies for Smoking Exposure

The microbial etiology of periodontal disease has been the focus of attention over the years, and various hypotheses have been proposed (Hajishengallis and Lamont, 2012). Notwithstanding, *Porphyromonas gingivalis* (*P. gingivalis*), a black-pigmented Gram-negative asaccharolytic bacterium from the phylum *Bacteroidetes*, has long been considered as an important pathogen involved in the initiation and progression of periodontal disease. *P. gingivalis* was shown to have a profound effect on both the amount and composition of the oral microbiota, even at a low abundance by acting as a potential community activist (Hajishengallis et al., 2011; Darveau et al., 2012). With a variety of virulence factors (such as the well-known gingipains), *P. gingivalis* can manipulate the host immune response by different strategies, finally leading to periodontal disease (Zenobia and Hajishengallis, 2015; Sochalska and Potempa, 2017). Therefore, the *in vitro* effects of smoking have mostly been investigated using *P. gingivalis* as a model pathogen (Zhou et al., 2007; Bagaitkar et al., 2009; Bondy-Carey et al., 2013).

The smoke generated upon the burning of tobacco is a complex, dynamic, and reactive mixture of over 5000 chemicals, with cytotoxic, mutagenic, carcinogenic, or antigenic properties (Talhout et al., 2011). Nicotine, a potent parasympathomimetic alkaloid, is the most well-known constituent with a highly addictive nature. It is considered as a major contributor to the development of dependence and is responsible for the widespread use and difficulty of quitting smoking (Pollock et al., 2009). Therefore, nicotine and its major metabolite cotinine have been widely used to investigate the influence of smoking on periodontal microorganisms (Cogo et al., 2009; Baek et al., 2012). Besides, cigarette smoke extract (CSE) and cigarette smoke condensate (CSC) are also representative cigarette smoke solutions for conducting the *in vitro* biological test, in which the non-nicotine constituents are considered (Zhang et al., 2010; Imamura et al., 2015).

#### Effects of Smoking on the Growth and Virulence of Key Pathogens

Cogo et al. (2008) tested the effects of nicotine and cotinine on growth of seven oral bacteria species including *P. gingivalis* at concentrations of 400, 100, 25, 6.25, 1.5, and 0.4 μg/mL, which were in agreement with or higher than the physiological levels of nicotine and cotinine found in saliva and gingival crevicular fluid (McGuire et al., 1989; Chen et al., 2001; Dhar, 2004); however, nicotine and cotinine did not alter the growth patterns of any of the bacteria tested. Similarly, the growth of *P. gingivalis*, *Fusobacterium nucleatum* (*F. nucleatum*), and *Filifactor alocis* (*F. alocis*) was also shown to be unaffected by CSE exposure at concentrations of 0.5, 2, and 4 μg/mL nicotine equivalents (Bagaitkar et al., 2009; Zeller et al., 2019). Bacteria are equipped with sophisticated mechanisms for adapting to complex environmental changes and thereby ensuring adequate growth and survival within the host (Brooks et al., 2011). Consistent with this fact, while the growth of periodontal pathogens is not found to be directly influenced by smoking, some changes in the virulence factors of bacteria are observed (Table 1). CSE changed the phenotype of *P. gingivalis* by up-regulating the gene expression of major fimbrial antigen (FimA), inducing the protein expression of the outer membrane RagA and RagB, suppressing the production of capsular polysaccharides, and neutralizing the proinflammatory response to subsequent TLR2 stimulation (Bagaitkar et al., 2009). Nevertheless, most effects were reversed when CSE-exposed bacteria were sub-cultured in a fresh medium without CSE. Thus, *P. gingivalis* appears to reversibly respond to CSE as an environmental stress. CSE also influenced the cell-bound Kgp and RgP gingipain production in a strain-specific manner (suppression in *P. gingivalis* ATCC33277 but augmentation in *P. gingivalis* W83) (Bondy-Carey et al., 2013). In addition, CSE exposure decreased the proinflammatory capacity (TNF-a, IL-6) of *P. gingivalis* biofilms (Bagaitkar et al., 2011). Taken together, these studies suggest that periodontal pathogens can withstand the complex mixture of toxins in smoking by altering their virulence factors.

**TABLE 1:** Effects of smoking on the virulence factors of key pathogens.

**References**	**Stimulus**	**Pathogens**	**Principle findings**
Baek et al., 2012	Nicotine	*Pg*	Nicotine exposure changed the expression pattern of low molecular weight proteins of *Pg*.
Zeller et al., 2019	CSE	*Pg*, *Ff*, *Fn*	CSE exposure changed the short chain fatty acid production.
Bagaitkar et al., 2010	CSE	*Pg*	CSE exposure up-regulated *Pg* FimA, suppressed the production of capsular polysaccharides, and created conditions that promote biofilm formation.
Bagaitkar et al., 2009	CSE	*Pg*	CSE exposure altered gene expression (such as oxidative stress-related and DNA repair genes) and outer membrane proteins (such as virulence factors RagA and RagB) of *Pg*.
Bondy-Carey et al., 2013	CSE	*Pg*	CSE exposure influenced cell-bound *Pg* Kgp and Rgp gingipain production in a strain-specific manner.
Bagaitkar et al., 2011	CSE	*Pg*, *Sg*	CSE exposure increased *Pg* FimA total protein, promoted *Pg*-*Sg* dual species biofilm formation, and decreased the pro-inflammatory capacity (TNF-α, IL-6) of *Pg* biofilms.

*CSE, cigarette smoke extract; *Pg*, *Porphyromonas gingivalis*; *Ff*, *Filifactor alocis*; *Fn*, *Fusobacterium nucleatum*; *Sg*, *Streptococcus gordonii*.*

#### Effects of Smoking on the Microbial Functions of Pathogen–Host Cell Interaction

The effects of smoking on the microbial functions of pathogen–host cell interaction constitute another major part of the *in vitro* studies (three main research focuses are summarized in Table 2). The adherence and invasion of bacteria to the host cells are important steps in the pathogenesis of infections. Epithelial cells that encompass mucosal surfaces represent the first line of defense against bacterial colonization (Tribble and Lamont, 2010). A few studies have investigated the ability of periodontal pathogens to colonize the epithelial cells after bacterial or cellular exposure to harmful substances from smoking (Teughels et al., 2005; Cogo et al., 2009; Imamura et al., 2015); however, discrepant results were reported. Teughels et al. (2005) tested the susceptibility of primary human gingival epithelial cells to be colonized by *P. gingivalis* and *Actinobacillus actinomycetemcomitans* (*A. actinomycetemcomitans*) after pre-exposing the cells to 1 mg/mL cotinine or nicotine. A species-dependent effect of nicotine and cotinine was observed. Whereas a moderate increase in attachment to cells was found in *A. actinomycetemcomitans*, a decreased attachment was recorded in *P. gingivalis*. On the contrary, the study by Cogo et al. (2009) showed that 100 μg/mL cotinine increased the adherence and invasion of *P. gingivalis* to epithelial cells (KB cells), when the bacteria were exposed to this substance; the pre-exposure of epithelial cells to cotinine or nicotine did not alter the colonization properties of *P. gingivalis*. Another study reported that a low concentration (1 μg/mL) of CSC increased the invasion of *P. gingivalis* to human gingival epithelial cells (Ca9-22) only both bacterial and cellular exposure were performed (Imamura et al., 2015). Therefore, due to the differences in the experiment design between different studies, such as the concentrations of the materials and cells tested, no certain conclusion could be drawn based on current studies regarding the possible correlation between bacterial cell colonization of periodontal pathogens and smoking exposure.

**TABLE 2:** Summary of *in vitro* studies of the effects of smoking on microbial functions for pathogen–host interaction.

**Research focus**	**Principle findings (references)**
Bacterial colonization and invasion	Bacterial cotinine exposure increased the association and invasion of *Pg* to human epithelial cells (KB cells) (Cogo et al., 2009).
	Cellular nicotine and cotinine exposure altered the colonization of *Aa* and *Pg* to primary human gingival epithelial cells in a species-dependent manner (Teughels et al., 2005).
	CSC increased the invasion of *Pg* to human gingival epithelial cells (Ca9-22) only both bacterial and cellular exposure were performed (Imamura et al., 2015).
Cytokine production/inflammatory response	Nicotine and *Pg* LPS synergistically upregulated IL-6 and IL-8 production in HGFCs (Wendell and Stein, 2001).
	Combination of nicotine and *Pg* LPS altered GRO-a, IL-7, IL-10, IL-15, RANTES, and IFN-g expression in HGFCs (Almasri et al., 2007).
	Nicotine and *Pg* LPS synergistically induced the inflammatory effects by inducing NO and PGE_2_ production, and increasing iNOS, COX-2, and HO-1 protein expression in HPDLCs (Pi et al., 2010).
	Nicotine and cotinine reduced superoxide responses of neutrophils stimulated with *Pg* LPS (Matthews et al., 2012).
	Nicotine inhibited the inflammatory response of HUVECs stimulated with *Pg* LPS (An et al., 2014).
Collagen degradation/periodontal tissue destruction	Combined effects of CSC and *Pg* increased HGFCs-mediated collagen degradation by destroying the balance between MMPs and TIMPs (Zhang et al., 2010).
	Nicotine and *Pg* had an addictive effect on HGFCs-mediated collagen degradation by influencing MMPs and TIMPs production (Zhou et al., 2007).
	Nicotine and *Pg* LPS promoted periodontal tissue destruction by inducing PGE_2_, MMP-2, and MMP-9 productions, and increasing MMP-2, MMP-9, COX-2, and HIF-1α protein expressions in HPDLCs (Kim et al., 2012).
	Nicotine and *Pg* LPS stimulated alveolar bone resorption by increasing MMPs and tissue-type PA expression in osteoblast (Katono et al., 2009).

*LPS, lipopolysaccharide; CSE, cigarette smoke extract; CSC, cigarette smoke condensate; PA, plasminogen activator; HGFCs, human gingival fibroblast cells; HPDLCs, human periodontal ligament cells; HUVECs, human umbilical vein endothelial cells; *Pg*, *Porphyromonas gingivalis*; *Aa*, *Aggregatibacter actinomycetemcomitans*.*

Nicotine and *P. gingivalis* lipopolysaccharide (LPS) were shown to influence the inflammatory response by altering the cytokine production and interfering with immune cell functions (Wendell and Stein, 2001; Almasri et al., 2007; Pi et al., 2010; Matthews et al., 2012). However, such inflammatory effects could be attenuated by carbon monoxide-releasing molecule-3 via the heme oxygenase-1 (HO-1) pathway (Song et al., 2017) or by interfering with the function of resistin (Kang et al., 2015). High concentrations of nicotine also inhibited the inflammatory response of human umbilical vein endothelial cells stimulated by *P. gingivalis* LPS, which might be associated with decreased gingival bleeding indices in smoking periodontitis patients (An et al., 2014). Similarly, as an explanation to more severe periodontal destruction in smokers compared to non-smokers, the combination of nicotine and *P. gingivalis* or *P. gingivalis* LPS increased collagen degradation and bone resorption by tipping the balance between matrix metalloproteinases (MMPs) and tissue inhibitors of metalloproteinases (TIMPs) (Zhou et al., 2007; Katono et al., 2009; Zhang et al., 2010; Kim et al., 2012).

### Clinical Findings of Subgingival Microflora in Smokers

Given the convincing evidence for differences in the clinical and immunological statuses of the subgingival environment in smokers and non-smokers, it would be reasonable to propose that the subgingival microflora should also exhibit differences between these two types of subject. However, data on the effect of smoking on subgingival microflora are inconsistent in early studies. Some of them found no difference in the subgingival microflora between smokers and non-smokers with different periodontal conditions, concluding that smoking had insignificant effects on the subgingival microflora (Darby et al., 2000; Bostrom et al., 2001; Van der Velden et al., 2003; Apatzidou et al., 2005; Natto et al., 2005; Salvi et al., 2005). In contrast, others reported a higher prevalence of periodontitis and periodontal pathogen counts in smokers, depending on the quantity and duration of cigarette smoking (Shiloah et al., 2000; Haffajee and Socransky, 2001; Gomes et al., 2006). The conflicting findings in these studies might be partly explained by the sensitivity and specificity of the microbiological methods used, including culture (Van der Velden et al., 2003), DNA probes (Shiloah et al., 2000), polymerase chain reaction (PCR) (Darby et al., 2000; Apatzidou et al., 2005), real-time PCR (Gomes et al., 2006), and DNA–DNA hybridization (Bostrom et al., 2001; Haffajee and Socransky, 2001; Natto et al., 2005; Salvi et al., 2005). The difference between smokers and non-smokers in probing depth could also be an explanation as smokers have deeper periodontal pockets than non-smokers, which might confound the effect of smoking on periodontal pathogens (Kigure et al., 1995).

**FIGURE 1:**
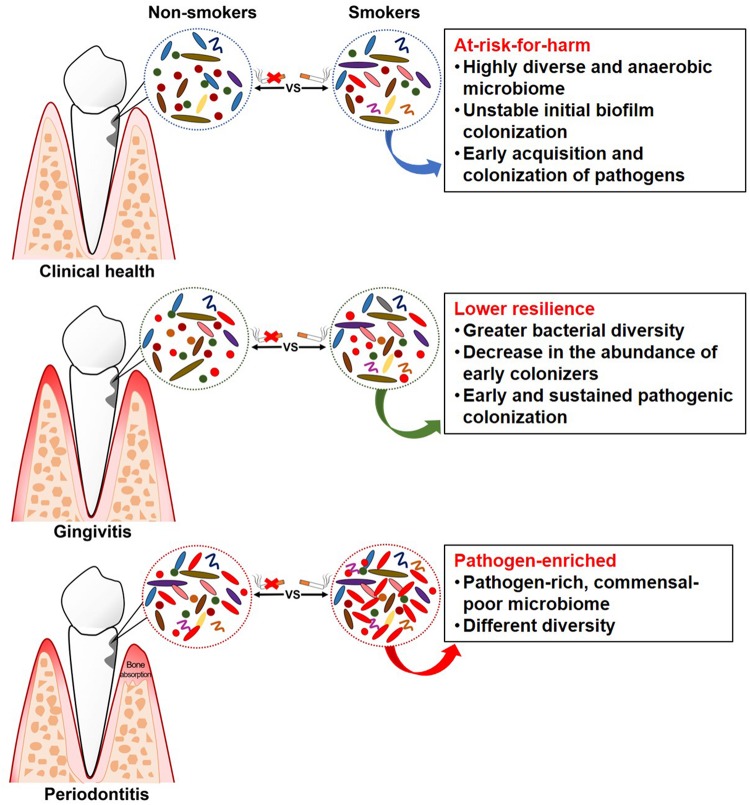
Schematic summary of clinical findings of subgingival microflora in smokers.

As it is now known that subgingival microflora is far more diverse than what was previously suspected (Griffen et al., 2012), and combined with the limitations of traditional targeted molecular methods (mentioned above) for bacterial identification, it was unknown previously whether smoking can cause qualitative and quantitative changes in the subgingival microflora. A novel technology for microbiome detection by 16S sequencing has been applied to identify the association of smoking and subgingival microflora during the past few years, opening up new horizons for a comprehensive understanding of whether and how these communities are affected. Table 3 presents a summary of the clinical studies (published between 2010 and 2019) on the effects of smoking on subgingival microflora using subgingival plaque samples from subjects with different periodontal conditions. Studies combining systemic conditions (such as diabetes mellitus and pregnancy) (Paropkari et al., 2016; Ganesan et al., 2017; Joaquim et al., 2018) or using granulation tissue from the subgingival area as samples (Coretti et al., 2017; Chowdhry et al., 2018) were also found but were excluded in this review. As shown in Table 3 and Figure 1, while contradictory results were reported by studies employing traditional targeted molecular methods (Kubota et al., 2011; Heikkinen et al., 2012; Guglielmetti et al., 2014; Lanza et al., 2016; Karasneh et al., 2017), altered subgingival microbial communities due to smoking in different periodontal conditions were generally revealed using 16S sequencing (Bizzarro et al., 2013; Mason et al., 2015; Yu et al., 2017).

**TABLE 3:** Clinical studies in relation to the effects of smoking on subgingival microflora (published between 2010 and 2019).

**References**	**Periodontal condition involved**	**Laboratory techniques**	**Microorganisms targeted**	**Main results (smokers vs. non-smokers)**
Heikkinen et al., 2012	/	PCR	*Aa*, *Pg*, *Tf*, *Pi*, *Pn*, *Td*	Higher prevalence of *Pi*, *Tf*, and *Td* in female smokers.
Kubota et al., 2011	CP	PCR	*Aa*, *Pg*, *Pi*, *Tf*, *Fn*/*Fp*, *Td*, *Cr*	Higher prevalence of *Cr* and lower prevalence of *Aa* in smokers.
Guglielmetti et al., 2014	CP	Quantitative PCR	*Aa*, *Pg*, *Tf*, *Td*	Greater amounts of *Pg*, *Aa*, and *Tf* in smokers; a significant association between smoking and the presence of *Aa*.
Karasneh et al., 2017	Healthy, CP	PCR	25 species	No difference between smokers and non-smokers in healthy status; higher *Ta* in smokers with periodontitis.
Lanza et al., 2016	CP, AP	High pure PCR	*Pg*, *Tf*, *Td*, *Pi*, *Aa*	No difference.
Bizzarro et al., 2013	Moderate to severe CP	Culture	*Aa*, *Pg*, *Pi*, *Tf*, *Pm*, *Fn*, *Cr*	No difference.
		Quantitative PCR	*Aa*, *Pg*, *Pi*, *Tf*, *Pm*, *Fn*, *Td*	No difference.
		16S sequencing	Community	Higher abundance of *Fusobacterium*, *Prevotella*, and *Selenomonas* in smokers; one cluster was identified by PCoA composed mainly of smokers (80%) with lower taxonomic diversity.
Mason et al., 2015	Healthy	16S sequencing	Community	Microbial profiles of smokers and non-smokers were different at all taxonomic levels; a highly diverse, pathogen-rich, commensal-poor, and anaerobic microbiome in smokers.
Kumar et al., 2011	Healthy	16S sequencing	Community	A highly diverse and relatively unstable initial colonization of subgingival biofilms in smokers, with more periodontal pathogens of *Fusobacterium*, *Cardiobacterium*, *Synergistes* and *Selenomonas*.
Yu et al., 2017	Healthy	16S sequencing	Community	Microbial diversity and composition were not significantly different by smoking status.
Joshi et al., 2014	Gingivitis, healthy	16S sequencing	Community	An early pathogenic colonization that led to sustained pathogen enrichment with periodontal pathogens in the biofilm, and lower resilience of the ecosystem in smokers.
Shchipkova et al., 2010	Moderate to severe CP	16S sequencing	Community	Greater abundance of *Parvimonas*, *Fusobacterium*, *Campylobacter*, *Bacteroides*, and *Treponema* and lower levels of *Veillonella*, *Neisseria*, and *Streptococcus* in smokers.
Moon et al., 2015	Moderate CP	16S sequencing	Community	Higher abundance of *Fusobacterium*, *Fretibacterium*, *Streptococcus*, *Veillonella*, *Corynebacterium*, and *Filifactor*, and greater bacterial diversity in smokers.
Camelo-Castillo et al., 2015	CP	16S sequencing	Community	Higher prevalence of *Granulicatella* and lower bacterial diversity in smokers.

*Studies related to smoking cessation and periodontal treatment are not included in this table (discussed in separate parts). Literature search was performed from the year 2000, but no 16S sequencing related study was found between 2000 and 2009. Besides, no additionally valuable information could be extracted from these reports apart from the main results listed in this table. Therefore, only papers published between 2010 and 2019 were summarized in this table. CP, chronic periodontitis; AP, aggressive periodontitis; PCR, polymerase chain reaction; PCoA, principal coordinate analysis. *Aa*, *Aggregatibacter actinomycetemcomitans*; *Pg*, *Porphyromonas gingivali*s; *Tf*, *Tannerella forsythia*; *Pi*, *Prevotella intermedia*; *Pn*, *Prevotella nigrescens*; *Td*, *Treponema denticola*; *Fn/Fp*, *Fusobacterium nucleatum/periodonticum*; *Cr*, *Campylobacter rectus*; *Ta*, *Treponema amylovorum*.*

#### Clinical Health

Studies have revealed some “unhealthy” signatures in the subgingival microflora of smokers even without any clinical manifestation. Mason et al. (2015) collected the subgingival plaque samples from 200 systemically and periodontally healthy smokers and non-smokers, and the microbial profiles were analyzed by 16S pyrotag sequencing. Two clusters with distinct microbial composition were revealed by principal coordinate analysis (PCoA), which were mainly composed of either smokers or non-smokers. The subgingival microflora of smokers was characterized by a highly diverse, pathogen-rich, commensal-poor, and anaerobic microbiome, which is thought to be more closely related to a disease-associated community. Moreover, periodontally healthy smokers had a highly diverse and relatively unstable initial colonization of subgingival biofilms, and an early acquisition and colonization of periodontal pathogens belonging to the genera *Fusobacterium*, *Cardiobacterium*, *Synergistes*, and *Selenomonas* (Kumar et al., 2011). Therefore, smoking may play a role in creating an “at-risk-for-harm” subgingival microbial community, making the subgingival environment more prone to periodontal destruction.

#### Gingivitis

Gingivitis is a necessary precursor to periodontitis, and the experimental gingivitis model provides a controlled and reversible method for examining the dynamic changes in health and disease (Loe et al., 1965). A longitudinal study, which was carried out on subjects with pre-existing gingivitis following a subsequent gingivitis resolution, experimental gingivitis induction, and experimental gingivitis resolution strategy, examined and compared the subgingival microflora in smokers and non-smokers at different clinical stages (Joshi et al., 2014). In this study, smokers demonstrated greater subgingival bacterial diversity than non-smokers during naturally occurring gingivitis and resolution state from disease to health. Smokers also had an earlier onset of clinically visible inflammation compared to non-smokers, which was attributed to the early pathogenic colonization, leading to sustained pathogen enrichment with periodontal pathogens. This is consistent with other findings in smokers, indicating that gingivitis is preceded by a decrease in the abundance of early colonizers, such as the genera *Streptococcus* and *Veillonella*, and an increase in the abundance of periodontopathogens, such as the genera *Treponema* and *Selenomonas* (Matthews et al., 2013; Peruzzo et al., 2016). The capability of an ecosystem to deal with perturbations while maintaining itself in a steady state in which the core species and key functions are retained is defined as resilience, which plays an important role in susceptibility to disease (Rosier et al., 2018). However, smokers demonstrated higher abundance of pathogenic species, suggesting that the subgingival microflora in smokers had lower resilience as they failed to maintain themselves in the original state when dealing with perturbation, thereby possibly increasing the risk to future disease (Joshi et al., 2014).

#### Periodontitis

Evidence has steadily accumulated, suggesting a pathogen-enriched community in smokers compared to non-smokers in periodontitis. Bizzarro et al. (2013) compared the subgingival microbiome in smokers and non-smokers with chronic periodontitis, using two traditional targeted techniques and a next-generation sequencing method. While no differences were found in the prevalence and quantity for any of the targeted species (Table 3) between smokers and non-smokers with culture and quantitative PCR, different subgingival microbial profiles were identified by pyrosequencing. Operational taxonomic units (OTUs) belonging to the genera *Fusobacterium*, *Prevotella*, and *Selenomonas* were more abundant in smokers, while OTUs belonging to the genera *Peptococcus* and *Capnocytophaga* were more abundant in non-smokers. Differences in bacterial communities between smokers and non-smokers were also detected at different taxonomic levels in other studies (Shchipkova et al., 2010; Camelo-Castillo et al., 2015; Moon et al., 2015), with variations in the types of bacteria identified. At the general level, *Fusobacterium*, as the most frequently identified bacteria, was more abundant in smokers than non-smokers, which was suggested to be one of the major determinants of subgingival bacterial community shift induced by smoking (Moon et al., 2015). *Fusobacterium*, especially the *F. nucleatum*, plays a critical role in the subgingival biofilm due to its “bridging species” role among microorganisms as well as its local immunosuppressive capability (Signat et al., 2011), thus contributing to the progression and severity of periodontal disease. Other bacteria that are consistently associated with periodontal disease include *Parvimonas*, *Treponema*, *Filifactor*, and *Bacteroides* (Kumar et al., 2005; Shi et al., 2015). However, conflicting results regarding the abundance of two common genera, i.e., *Streptococcus* and *Veillonella*, were reported in two studies (Shchipkova et al., 2010; Moon et al., 2015). As *Veillonella* and *Streptococcus* species are known to be abundant in health-associated biofilms (Paster et al., 2001; Kumar et al., 2005, 2006), the higher abundance in smokers found in the study by Moon et al. (2015) was speculated to be the result of bacterial interaction.

Considering the microbial diversity, which is thought to be another important parameter for an ecosystem, different results are reported in different studies. Bizzarro et al. (2013) identified one cluster by PCoA, which was mainly composed of smokers (80%) with significantly lower taxonomic diversity, consistent with the findings of a study by Camelo-Castillo et al. (2015). However, some studies have reported a higher diversity in smokers compared to non-smokers (Moon et al., 2015), or no difference in the mean number of species/phylotypes between smokers and non-smokers (Shchipkova et al., 2010). As microbial diversity in a given microbial community not only refers to the species richness but also relates to their evenness, the different conclusions in recent studies might be attributed to the different indices used to evaluate microbial diversity. Furthermore, the bioinformatic data processing method of the obtained sequences can also influence the results (Nearing et al., 2018). Although the important role of key species in disease development has been recognized, the role of microbial diversity is less clear (Karkman et al., 2017). Some studies have shown a higher microbial diversity at more severely affected sites in periodontitis (Griffen et al., 2012), but some others have reported it the other way around (Kirst et al., 2015). Taking these findings together, it seems that no unified conclusion can be drawn currently regarding how smoking-related microbial diversity changes would contribute to periodontitis.

### Effect of Smoking Cessation on the Subgingival Microflora

Using a quantitative and cultivation-independent method, i.e., terminal restriction fragment length polymorphism for bacterial profiling, Fullmer et al. (2009) analyzed the subgingival plaque samples from smokers and quitters and showed that microbial profiles differed significantly between these two groups at 6- and 12-month intervals after giving up smoking. The microbial community in smokers was similar to baseline, while quitters exhibited significantly divergent profiles. At the bacterial species level, smoking cessation led to a decrease in periodontal pathogens, including *Porphyromonas endodontalis*, *Dialister pneumosintes*, *Parvimonas micra*, *F. alocis*, and *Treponema denticola* (*T. denticola*), in association with an increase in the level of health-associated species *Veillonella parvula* (Delima et al., 2010). The beneficial effects of smoking cessation on the periodontium are evident. Smoking cessation reduces the risks of the onset and progression of periodontal disease, reduces the risk of tooth loss, and improves the clinical outcomes of periodontal therapy (Dietrich et al., 2015; Leite et al., 2018). From the microbiological point of view, previous studies have also revealed the crucial role of smoking cessation in changing the subgingival microflora, and consequently, the response to periodontal treatment.

### Effect of Smoking on Subgingival Microflora in Periodontal Treatment

Since smokers have different etiologies and clinical manifestations of periodontal diseases compared to non-smokers, it is not surprising that they also respond differently to periodontal treatment. The negative influence of smoking on the response to periodontal therapies has been reviewed previously through clinical parameters, such as bleeding on probing (BoP) and probing pocket depth (PD) (Heasman et al., 2006; Nociti et al., 2015). Most recently, some microbiological findings concerning periodontal treatment response in smokers additionally suggest the adverse effects of smoking. Although the clinical parameters improved in both smoking and non-smoking periodontitis patients following non-surgical and/or surgical therapy, a lower reduction and greater post-therapy prevalence of periodontal pathogens, including the well-known “red-complex” and “orange-complex” bacteria, were observed in smokers (van Winkelhoff et al., 2001; Van der Velden et al., 2003; Darby et al., 2005; Meulman et al., 2012; Bunaes et al., 2015). Smokers were also reported to be more susceptible to the re-establishment of a pathogenic subgingival biofilm than non-smokers after scaling and root planning (SRP) because a significant decrease in the pathogenic species was only observed in non-smokers 180 days after treatment (Feres et al., 2015).

It is therefore rational to utilize adjunctive antimicrobial agents, either locally or systemically, in the periodontal treatment of smokers based on the evidence that subgingival pathogens seem to be more difficult to eliminate in smokers following non-surgical and surgical therapy. Machion et al. (2004) showed that locally delivered doxycycline promoted the elimination of *Tannerella forsythia* (*T. forsythia*) and *P. gingivalis* in a greater proportion of sites compared to conventional SRP in smokers with chronic periodontitis. Similarly, SRP alone was ineffective in reducing the counts or proportions of the “red-complex” or “orange-complex” bacteria in current smokers with periodontitis, whereas a combination of minocycline and SRP significantly reduced both (Grossi et al., 2007). The adjunctive use of metronidazole and amoxicillin systemically in the SRP treatment of smokers with chronic periodontitis also led to the most beneficial changes in the subgingival microbial profile by reducing the mean counts and proportions of periodontal pathogens, such as *T. forsythia*, *P. gingivalis*, and *T. denticola*, and increasing the proportions of host-compatible species (Matarazzo et al., 2008; Faveri et al., 2014). The microbiological effect of adjunctive antimicrobial photodynamic therapy on non-surgical periodontal treatment was also investigated; however, no difference was identified (Queiroz et al., 2014).

The use of dental implants has become a popular alternative for the replacement of missing teeth. Several studies have evaluated the influence of smoking on the peri-implant microflora, demonstrating a trend similar to the microbiological findings in the subgingival microflora, indicating that smoking is associated with a higher prevalence of pathogenic species (Tsigarida et al., 2015; Ata-Ali et al., 2016; Eick et al., 2016; Stokman et al., 2017). The most impressive findings were presented by a study using a deep-sequencing method to identify the effect of smoking on the peri-implant microbiome in the health and disease states (Tsigarida et al., 2015). Compared to non-smokers, microbial signatures of health in smokers exhibited a lower diversity and an enrichment in species traditionally regarded as periodontal and/or systemic pathogens, including those belonging to the genera *Capnocytophaga*, *Treponema*, *Propionibacterium*, *Pseudomonas*, *Lactobacillus*, and *Leptotrichia*. Although a core microbiome is identified, which is composed of species most suited to the peri-implant habitat in both smokers and non-smokers, smoking modifies this environment containing 31 different species. The transition of the microbiome from health to mucositis has also been observed to be different in smokers and non-smokers, although peri-implant mucositis is an important event in both groups. In non-smokers, it resembles primary ecological succession, with the acquisition of several species without replacement of pioneer organisms; however, in smokers, further enrichment in pathogenic species and a decrease in diversity are shown.

## Potential Mechanisms Contributing to the Negative Impact of Smoking on Subgingival Microflora

**FIGURE 2:**
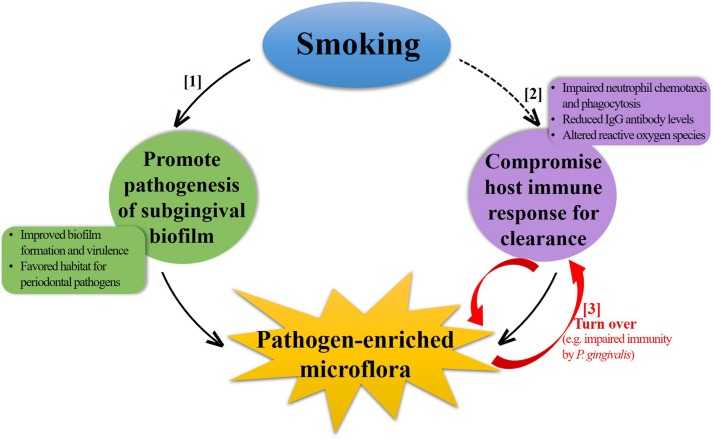
Schematic illustration of potential mechanisms contributing to the negative impact of smoking on subgingival microflora. Smoking can induce a pathogen-enriched subgingival microflora through increasing the pathogenesis of subgingival biofilm ([1]) and decreasing the host immune response for bacterial clearance ([2]). Some periodontal pathogens can also subvert the host immune response in turn, forming a vicious circle ([3]).

Subgingival microflora is a highly diverse and structured community attaching to the tooth surface as a biofilm. A comprehensive understanding of the underlying mechanisms by which smoking influences these biofilms is challenging with currently limited studies, as in addition to the direct effects of smoking on biofilm formation and succession, an indirect effect, mainly from the host immune response, must also be taken into account under the subgingival settings. Furthermore, the complex interplay between these two aspects makes it more complicated. For a brief discussion here on several possible mechanisms (Figure 2), the situation is simplified into two separate parts by the dominant factor discussed.

### Promote Pathogenesis of Subgingival Biofilm (Figure 2, [1])

Biofilm formation in the oral cavity is a gradated process, consisting of four stages, of which the initial bacterial adhesion and colonization are crucial steps (Hao et al., 2018). In this regard, smoking can enhance biofilm formation by some key species. The *in vitro* effects of smoking on key pathogens (as reviewed in the section “*In vitro* Effects of Smoking on Key Periodontal Pathogens”) showed that exposure to CSE can alter the expression of several virulence factors of *P. gingivalis*, facilitating biofilm formation process by upregulating the expression of FimA and several outer membrane proteins (RagA, RagB, and a putative lipoprotein co-transcribed with the minor fimbrial antigen), and suppressing the expression of capsular polysaccharide (Bagaitkar et al., 2009, 2010). FimA is the major fimbrial antigen of *P. gingivalis* that plays a critical role in bacterial colonization and invasion to the periodontium through adhering to salivary proteins, extracellular matrix, eukaryotic cells, and bacteria of either the same or other species (Bostanci and Belibasakis, 2012). The observed upregulation of FimA not only increased the biofilm formation of *P. gingivalis* but also promoted the interaction between *P. gingivalis* and *Streptococcus gordonii* (an early biofilm colonizer) by improving its binding to glyceraldehyde-3-phosphate dehydrogenase, thus enhancing dual-species biofilm formation (Bagaitkar et al., 2011). Simultaneously, the upregulation of FimA also reduced the host response to *P. gingivalis* by abrogating the proinflammatory response to subsequent TLR2 stimulation (Bagaitkar et al., 2010), and therefore increasing bacterial survival. As *P. gingivalis* has a prominent role in orchestrating the virulence of the biofilm and the consequent inflammatory response, earning itself the characteristics of a “keystone” periodontal pathogen (Hajishengallis et al., 2012), a change in the virulence of *P. gingivalis* might exert a community-wide influence to promote the shift of subgingival biofilm to a dysbiotic state, predisposing the individual to periodontal destruction.

*Fusobacterium*, especially *F. nucleatum*, plays a key role in physical interactions between Gram-positive and Gram-negative species and is considered as an intermediate colonizer, bridging the attachment of commensals that colonize the tooth and epithelial surfaces with true pathogens (Signat et al., 2011). Thus, the frequently detected higher *Fusobacterium* amount in the subgingival microflora in smokers (Shchipkova et al., 2010; Bizzarro et al., 2013; Moon et al., 2015) might allow it to transport periodontal pathogenic bacteria, leading to a pathogen-enriched biofilm formation.

In addition to enhancing biofilm formation, some other phenomena might promote the pathogenesis of subgingival biofilm by creating a suitable environment for periodontal pathogens. For example, Hanioka et al. (2000) reported significantly low oxygen tension within the periodontal pockets in smokers, which might favor the growth of anaerobic periodontal pathogens even in shallow pockets; this was supported by the clinical findings that smoking creates a favorable habitat for bacteria, such as *P. gingivalis*, *Prevotella intermedia*, and *A. actinomycetemcomitans* at shallow sites (≤5 mm) (Eggert et al., 2001). Shah et al. (2017) showed that smoke-induced transcriptional shifts in commensal biofilms triggered a proinflammatory response and created a cytokine-rich, pro-oxidant, and anaerobic environment, leading to the early commensal death and creation of pathogen-enriched biofilms in smokers.

### Compromise Host Immune Response (Figure 2, [2])

Smoking compromises various aspects of the innate and adaptive host immune responses, as summarized in previous reviews of mechanisms of smoking-related periodontal destruction (Palmer et al., 2005; Ryder, 2007; Johannsen et al., 2014). The general observation is that smoking stimulates the inflammatory response and impairs protective response, thus accelerating the periodontal destruction. The changes associated with the immune response in subgingival environment can also have an impact on the microbial community. Neutrophils are the primary leukocytes, which are critical for the defense against bacterial invasion by phagocytosis in human body. However, both *in vitro* and *in vivo* studies have shown that smoking can impair the chemotaxis and phagocytosis of neutrophils in the periodontium (Guntsch et al., 2006; Zappacosta et al., 2011), leading to defective clearance of bacteria and thereby increasing the colonization. Furthermore, smoking has been shown to inversely correlate with the levels of serum immunoglobulin (Ig) G antibodies specific for some periodontal pathogens (Graswinckel et al., 2004; Tebloeva et al., 2014). Vlachojannis et al. (2010) assessed the levels of IgG antibody to multiple periodontal bacteria in a large population of US adults and found that current smokers were less likely to have higher antibody titers for periodontal pathogens, such as *P. gingivalis*, *Campylobacter rectus*, and *Prevotella nigrescens* after adjusting important confounding factors. The reduced level of IgG antibody can impair the host immune response and exert a “protective” effect on these periodontal pathogens. In addition, the generation of reactive oxygen species, which play an important role in intracellular bacterial killing, can be affected by smoking, possibly resulting in a decreased innate immune response to periodontal pathogens (Matthews et al., 2011). From another perspective, *P. gingivalis* can modulate innate host defense function, such as immune subversion of IL-8 secretion, complement activity, or TLR4 activation by several virulence factors, resulting in impaired host defense (Darveau et al., 2012). In this sense, it seems that a vicious circle (Figure 2, [3]) forms between pathogen-enriched subgingival microflora and the host immune response due to smoking, which might worsen the situation even further in terms of health.

## Conclusion

As discussed in this review, smoking exposure represents an environmental stress to which periodontal pathogens, such as *P. gingivalis*, can adapt by altering their gene and protein expressions. These changes may alter the virulence of bacteria and host–pathogen interactions, and finally contribute to the development of periodontal disease. Although early studies based on traditional targeted molecular methods yield conflicting findings concerning the effects of smoking on subgingival microflora associated with periodontal disease, microbial composition differences in smokers, compared to non-smokers, have been shown by the newly applied 16S sequencing technology, regardless of the periodontal condition. Smoking can create an “at-risk-for-harm” subgingival microbial community in the healthy periodontium, lower the resilience of the subgingival ecosystem in gingivitis, and promote a pathogen-enriched subgingival microflora in periodontitis, thus playing a role in the clinically observed increased susceptibility and severity of periodontal destruction in smokers. The pathogen-enriched subgingival microflora responds poorly to the periodontal treatment, whereas smoking cessation alters the subgingival biofilm, suggesting a mechanism for improved periodontal health associated with smoking cessation.

Although the negative impact of smoking on subgingival microflora is certain, the underlying mechanism is unclear and open for debate. In this review, the possible mechanisms by which smoking creates pathogen-enriched biofilms were discussed, including the promotion of pathogenesis of subgingival biofilms via key pathogens and the ineffective host immune response for clearance. However, we are currently just at the beginning when it comes to an understanding of the underlying mechanisms. Further studies are required to elucidate the mechanisms of the effect of smoking on subgingival microflora for the prevention of periodontal destruction.

## Author Contributions

YJ drafted the manuscript. XZ, LC, and ML edited and added the valuable insights into the manuscript. All authors approved the final manuscript and agreed to be accountable for all aspects of the work.

## Conflict of Interest

The authors declare that the research was conducted in the absence of any commercial or financial relationships that could be construed as a potential conflict of interest.

## Footnotes

1 https://www.who.int/news-room/fact-sheets/detail/tobacco
